# Spontaneous bleeding in COVID-19: A retrospective experience of an Italian COVID-19 hospital

**DOI:** 10.4102/sajr.v26i1.2509

**Published:** 2022-10-31

**Authors:** Mirko Trentadue, Plinio Calligaro, Gianluigi Lazzarini, Fabio Bonomi Boseggia, Elena Residori, Jennifer Hu, Silvana Vanti, Linda Lillo, Giovanna Varischi, Roberto Cerini

**Affiliations:** 1 Radiology Unit, M. Magalini Hospital, AULSS 9 Scaligera, Villafranca di Verona, Italy; 2 Intensive Care Unit, M. Magalini Hospital, AULSS 9 Scaligera, Villafranca di Verona, Italy; 3 Independent Researcher, Occupational Medicine Specialist, Peschiera del Garda, Italy; 4 Occupational Medicine Service, Palazzo della Sanità, AULSS 9 Scaligera, Verona, Italy; 5 Hospital Medical Management - District 4, Professional Staff Organization, AULSS 9 Scaligera, Villafranca di Verona, Italy

**Keywords:** COVID-19, bleeding, haemorrhage, haematoma, retroperitoneum

## Abstract

**Background:**

Haemorrhages in coronavirus disease 2019 (COVID-19) patients require proper knowledge and management.

**Objectives:**

To highlight the characteristics of haemorrhages in patients with COVID-19 infection.

**Method:**

A retrospective study examined CT scans performed over a 13-month period in patients hospitalised with COVID-19 infection to determine those who developed spontaneous bleeding. The authors also investigated correlations between the bleeding events and the patients’ characteristics.

**Results:**

Haemorrhages occurred in 2.22% (31/1396) of patients hospitalised with COVID-19 infection (7.88%, 19/241 in the intensive care unit). Bleeding, major in most cases, occurred in anticoagulated patients, especially males with multiple comorbidities, aged between 60 and 79 years and mainly appeared in a single anatomical region (especially retroperitoneal), with the most voluminous in the chest wall. The complication was diagnosed on average 16.7 days after admission and occurred predominantly in critically ill patients undergoing invasive ventilation and pronation-supination cycles. In just under half of the cases, the haematomas were active, and in these cases, mainly with a single contrast blush and with earlier onset after the start of anticoagulation than in non-active bleeding. Major bleeding was also earlier in the presence of multiple morbidity. The vast majority of patients were treated conservatively and survived.

**Conclusion:**

At COVID-19 hospital centres, it is advisable that there is knowledge of such a complication for which CT imaging is essential for diagnosis and proper management. Although some authors have expressed doubts about anticoagulant treatment in patients with COVID-19, the bleeding complication in this study did not significantly affect the outcome.

**Contribution:**

Spontaneous haemorrhage did not significantly affect the outcome in this series.

## Introduction

Haemorrhagic complications in coronavirus disease 2019 (COVID-19) patients require management referral from spoke facilities and biocontainment measures to central facilities (hub).^1^ CT imaging plays a pivotal role in the management of COVID-19 infection, particularly in critically ill patients. Since the onset of the severe acute respiratory syndrome coronavirus 2 (SARS-CoV-2) pandemic, CT has been the dominant modality in defining the severity of pulmonary involvement by allowing a rapid and preliminary stratification of patients into risk categories, providing useful elements for therapeutic planning and prognostic evaluation. CT imaging also has a fundamental role in the diagnosis and monitoring of complications, most of which are life-threatening, such as neurological, vascular, gastrointestinal or renal complications, thus improving the outcome of COVID-19 patients.^2,3^

This research describes the characteristics of spontaneous haematomas found on CT scans in a cohort of patients with COVID-19 infection, admitted to a COVID-19 hospital (‘M. Magalini’ Hospital, Villafranca di Verona - AULSS 9 Scaligera, Verona, Italy) and to search for any correlations between the bleeding events and any anamnestic, clinical and laboratory patient characteristics.

## Materials and methods

This was a single-centre retrospective observational study. The Radiology Information System-Picture Archiving and Communication System (RIS-PACS) database was analysed by examining all the CT scans performed from 01 March 2020 to 30 April 2021 in patients hospitalised with COVID-19 infection at ‘M. Magalini’ Hospital, Villafranca di Verona – AULSS 9 Scaligera, Verona, Veneto, Italy.

Male and female patients affected by COVID-19 (confirmed with molecular research of viral DNA using a nasopharyngeal swab) and who developed one or more haematomas during hospitalisation, were included. The CT examinations performed in both the unenhanced and post-contrast phases, involving at least one of the following body areas, were included in the evaluation: neck, thorax, abdomen, upper and lower limbs and pelvis. Any examinations targeting the spine, skull, and facial bones as well as those performed on patients with pathologies other than COVID-19 were excluded.

### Imaging, clinical, laboratory and therapeutic aspects

All studies were performed with a 16-layer multidetector CT scanner (TOSHIBA Astelion 16, Japan), 120 kV. The acquisition protocols involved an unenhanced phase, and when completed with intravenous iodenated contrast administration (iopromide, Ultravist 370 mg/mL, 1.2 mg/kg, Bayer HealthCare Pharmaceuticals Inc., Berlin, Germany), they were performed with bolus tracking, using an infusion flow of not less than 3.5 mL/s, with arterial (15–20 s), venous (60–70 s) and sometimes late (3–5 min) phase scans. The images were transmitted to the PACS for post-processing. Multiplanar reconstruction (MPR) and reprocessing was carried out with IMPAX Client software version 6.6.1.0 (AGFA HealthCare NV, Mortsel, Belgium).

Data recorded included whether the detected haematomas were single or multiple, noting the specific anatomical structures where the bleeding was located and grouping them into the following locations: thoracic wall, thoracic cavity, abdominal wall, retroperitoneum, intraperitoneal, pelvis, neck, upper and lower limbs. For each haematoma, the latero-lateral, craniocaudal and anteroposterior diameters were measured by means of MPR reconstructions, expressed in centimetres, approximating the measurements to the nearest whole number. The shape of the haematomas was qualitatively evaluated equivalent to ellipsoids, estimating their volume in cubic cm = mL with the formula:


V=4/3×πabc [Eqn 1]

using the online tool https://keisan.casio.com/exec/system/1223392149. For each haematoma, the presence of contrast blush, uni- or multifocal, in the arterial and venous phase was investigated.

Additional data included patient age and sex, hospitalisation date, discharge date, start of anticoagulant treatment date, length of hospitalisation, date of the first CT examination diagnostic for haematoma and number of days elapsed between this examination and the date of hospitalisation and initiation of anticoagulation, and whether during the hospitalisation they were admitted to the intensive care unit (ICU). These patients were standardised to receive enoxaparin twice a day (bid) based on body weight: 4000 U bid if < 80 kg, 6000 U bid if between 80 kg and 120 kg, 8000 U bid if > 120 kg. The enoxaparin dosage was modulated in relation to the D-dimer trend, creatinine clearance (< 30 mL/min), occurrence of bleeding and anaemia. All ICU patients who developed haematomas were transfused with at least one bag of concentrated red blood cells to ensure adequate oxygen delivery.

In cases with thrombocytosis (platelets [PLT] > 450*10^3^/µL), treatment with acetylsalicylic acid 75 mg/day – 100 mg/day was started, if not contraindicated.

In relation to the haemodynamic stability/instability and the blood count trend, a conservative approach was implemented with remodelling of the anticoagulant therapy using calcium heparin suspension of anti-aggregation and transfusion of multiple bags of red blood cells. If invasive management was necessary, the patients were transferred to the reference hub.

The data of patients who underwent invasive mechanical ventilation, reported trauma and major bleeding during hospitalisation, defined according to the criteria of the International Society on Thrombosis and Haemostasis (ISTH), were recorded.^4^

For each patient, previous comorbidities, any anticoagulant or antiplatelet treatments in place at the time of admission and some laboratory parameters measured at the time of both admission and the first diagnostic CT study were deduced from the electronic medical records. The appearance of other relevant complications, particularly of a thromboembolic ischaemic nature, diagnosed by CT, was also investigated. Finally, the outcome in patients discharged and deceased (from any cause) was recorded.

### Statistical analysis

The study utilised the IBM^®^ SPSS^®^ Statistics software package, version 28.0 (IBM Corp., Armonk, NY, United States [US]).

## Results

### Population

Among the 931 CT studies performed in the hospital from 01 March 2020 – 30 April 2021, the authors identified a cohort of 31 patients meeting the inclusion criteria (23 males [74.20%] and 8 females [25.80%]), 19 of whom (61.3%) were hospitalised in the ICU and 12 (38.7%) in non-intensive wards.

The average age was 71.12 years (range 52–93 years; males: 69.90, range 53–83 years; females: 74.11, range 52–93 years) for a total of 14 (45.16%) in the age group 70–79; 11 (35.48%) in the 60–69 age group; 3 (9.67%) in the 50–59 age group; 3 over 80 (9.67%): patients in the 60–79 age group represented 80.64% of the sample (25/31). There was a significant correlation (*p* = 0.01 with Pearson’s coefficient *r* = 0.44) between the male gender and the age group 60–79 years.

Bleeding occurred in 2.22% of patients hospitalised for COVID-19 (31/1396) while in the subpopulation of patients hospitalised in the ICU, the percentage was 7.88% (19/241). In 29/31 cases (93.6%), the bleeding was major according to ISTH criteria.

The average length of hospitalisation was 37.58 days (range: 9–138 days), with a mean of 42 days (range: 13–138) for patients admitted to ICU and 30.58 days (range: 9–48) for the others.

### Imaging

Haematomas were diagnosed on contrast CT scans in 30/31 cases (96.78%) while in 1/31 cases (3.22%) the diagnosis was reached by unenhanced CT imaging in a patient with reduced glomerular filtration rate; given the haemodynamic stability, a contrast medium injection was not necessary for the investigation.

The first CT diagnosis of haematoma was made on average at 16.70 days from hospitalisation (range 0–33 days), at 18.7 days in ICU patients (range 5–33) and at 13.6 days in patients admitted to other units (range 0–31 days). The diagnosis was made at admission in two patients.

**FIGURE 1:**
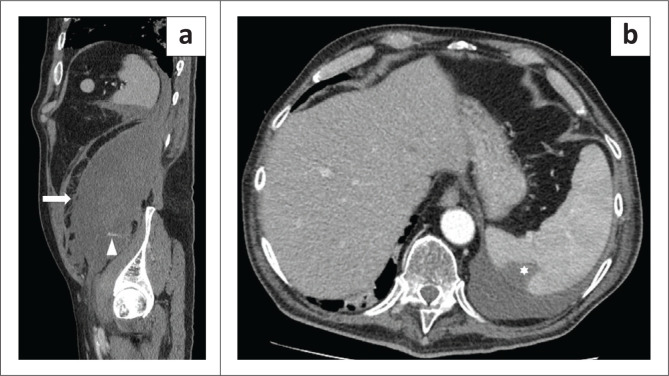
A 65-year-old man with a retroperitoneal haematoma in the left iliopsoas muscle, diagnosed 17 days after hospital admission. (a) Parasagittal plane, arterial phase: a contrast blush (arrowhead) can be seen in the lower part of the haematoma (arrow). (b) Axial plane, venous phase: the patient later developed a minor splenic infarct (asterisk). The patient underwent endovascular embolisation and survived.

**FIGURE 2:**
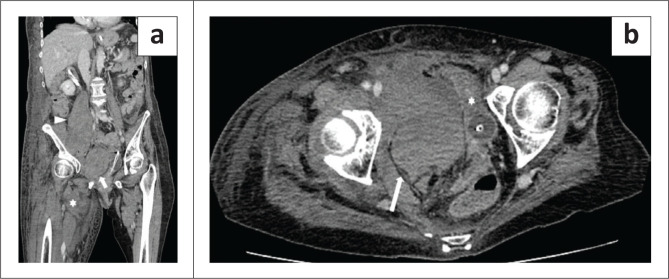
A 74-year-old woman with multiple haematomas, the first diagnosed 28 days after hospital admission. (a) Coronal plane, venous phase: the image shows the haematomas in the right iliopsoas muscle (arrowhead), in the right Retius’ space (arrow) and in the vastus medialis muscle of the right thigh (asterisk). (b) Axial plane, venous phase: the image shows the haematoma in the Retius’ space on the right side (arrow), displacing the bladder with urinary catheter inside the lumen (asterisk). The same patient later developed pulmonary thrombo-embolism (not shown).

**FIGURE 3:**
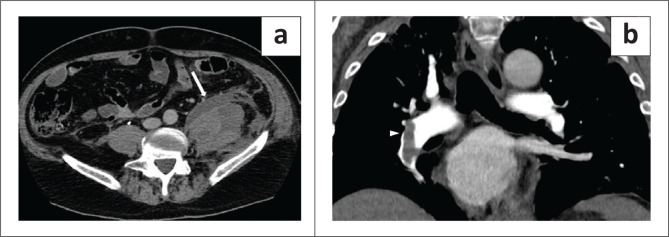
A 65-year-old man with multiple haematomas, the first diagnosed 22 days after hospital admission. (a) Axial plane, venous phase: haematoma (arrow) in the left psoas muscle. (b) Coronal plane, pulmonary arterial phase: endoluminal filling defect in the right pulmonary artery (arrowhead). The patient underwent endovascular treatment and survived.

**FIGURE 4:**
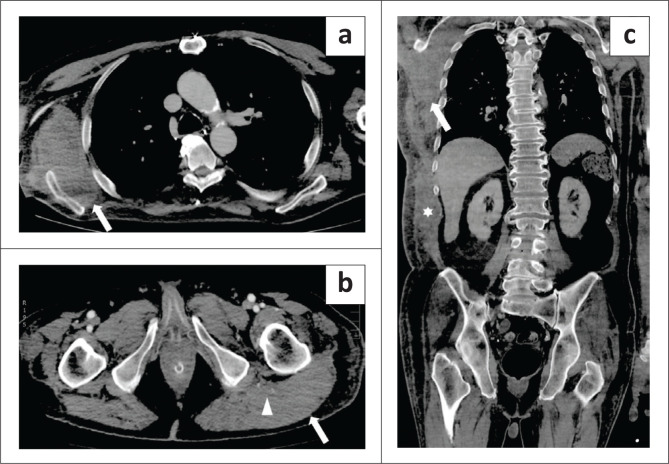
A 73-year-old man with multiple haematomas, the first diagnosed 18 days after hospital admission. (a) Axial plane, venous phase: right subscapular haematoma (arrow). (b) Axial plane, arterial phase: left gluteal haematoma (arrow) with a small blush in the inner part (arrowhead). (c) Coronal plane, venous phase shows the right subscapular haematoma (arrow) and a haematoma in the abdominal wall (asterisk).

**FIGURE 5:**
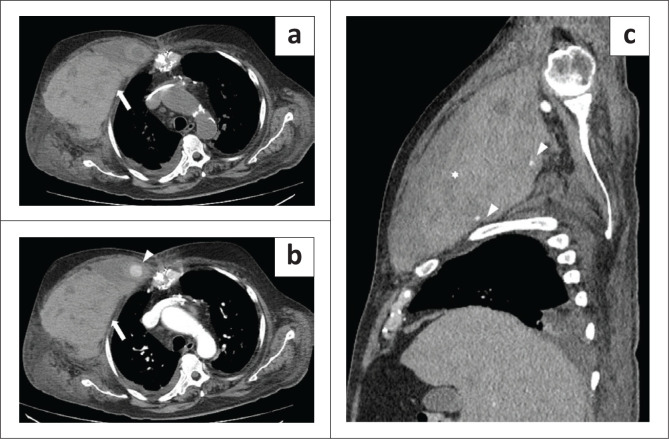
A 79-year-old man with a chest haematoma diagnosed 10 days after hospital admission. (a) Axial plane, not-enhanced phase demonstrates a huge inhomogeneous haematoma in the right chest wall (arrow). (b) Axial arterial phase image shows an active arterial bleed (arrowhead) in the medial part of the haematoma (arrow). (c) Parasagittal plane, arterial phase indicates the ellipsoid shape of the haematoma (asterisk). Some other small contrast blushes can be seen in the posterior part of the haematoma (arrowheads). The patient underwent endovascular embolisation and survived.

**FIGURE 6:**
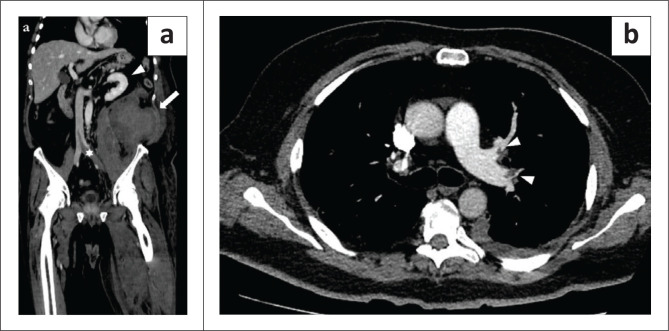
A 59-year-old man with a left retroperitoneal haematoma diagnosed 19 days after hospital admission. (a) Coronal plane, venous phase image shows a retroperitoneal haematoma (arrow) and an extensive thrombus in the left iliac vein extending into the inferior vena cava (asterisk). The left kidney is displaced by the haematoma (arrowhead). (b) Axial plane, pulmonary arterial phase demonstrates pulmonary thrombo-embolism (arrowheads). The patient underwent endovascular embolisation and survived.

**FIGURE 7:**
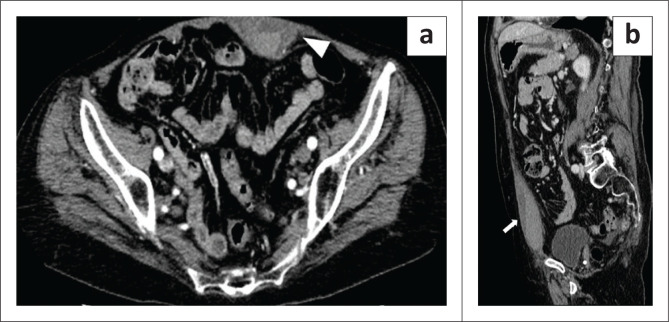
A 76-year-old woman with an abdominal wall haematoma diagnosed 8 days after hospital admission. (a) Axial arterial phase image reveals the haematoma in the left rectus muscle (arrowhead). No contrast blushes were detected. (b) Parasagittal venous phase image shows the longitudinal extent of the haematoma in the abdominal wall (arrow), with a layered fluid–fluid blood level. The same patient later developed pulmonary thrombo-embolism (not shown).

**FIGURE 8:**
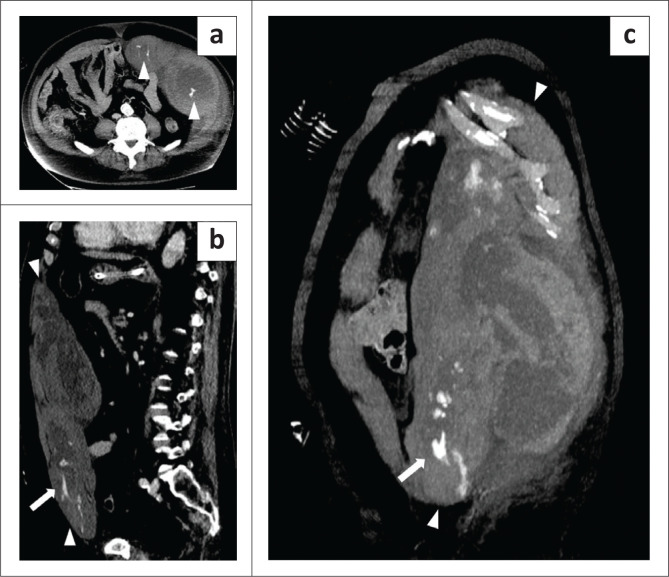
A 71-year-old man with a huge arterial feeder in an abdominal wall haematoma diagnosed 14 days after hospital admission. (a) Axial arterial phase, MIP reconstruction image demonstrates a bilobed-shaped left abdominal wall haematoma (arrow) with multiple foci of arterial blushes (arrowheads). (b) Arterial parasagittal and (c) coronal planes, MIP reconstruction images show the longitudinal extent of the haematoma (arrowheads) and the arterial blushes in its lower part (arrows). The patient, also developed pulmonary thrombo-embolism (not shown) and required endovascular embolisation twice, but later demised.

In all cases, unenhanced CT images revealed the presence of volumetric increase of the anatomical structures involved, which were inhomogeneously hyperdense (~ 60 Hounsfield Units). None of the cases raised differential diagnostic doubts with other pathologies (e.g. abscesses or sarcomas), given the clinical history (e.g. anaemia, hypotension, anticoagulant treatment) and imaging characteristics (e.g. absence of gas within the lesions, absence of calcifications or contrast enhancement, presence of contrast medium spillage) (Figures 1–8).

In 3/31 cases (9.67%) there was a history of trauma: two cases of accidental falls with minor injury and with an unclear correlation with bleeding diagnosed after a few days (retroperitoneal haematoma of 1900 mL, haematoma of 824 mL of the rectus femoris muscle), while in the third case, a small haematoma developed adjacent to the access site of a jugular central venous catheter. Iatrogenic significance was attributed to this bleeding (*p* = 0.001; *r* = 0.56), however, a retroperitoneal haematoma developed the next day without any trauma correlation.

In 22/31 patients (70.96%) the haematomas were single, while in 9/31 patients (29.04%), multiple haematomas were found with a total of 43 anatomical sites affected, listed in Table 1. There was a significant correlation between the presence of bleeding in the retroperitoneal space (*p* = 0.019; *r* = 0.42) and in the pelvis (*p* = 0.022; *r* = 0.41) and the development of multiple haematomas.

**TABLE 1:** Imaging characteristics.

Variable	Total (*n*)	%	Average (*n*)
**Patients**	31	-	
**Days since hospital admission to CT diagnosis**	-	-	16.7
**Major bleeding (ISTH)**	29	93.6	-
**History of trauma before diagnosis**	3	9.67	-
**Multiple haematomas**	9	29.04	-
**Bleeding sites**	43	-	-
Retroperitoneal	17	39.53	-
Volume (mL)	-	-	680
Abdominal wall	8	18.60	
Volume (mL)	-	-	749
Lower limbs	6	13.95	-
Volume (mL)	-	-	360
Chest wall	5	11.62	-
Volume (mL)	-	-	1061
Upper limbs	3	6.97	-
Volume (mL)	-	-	338
Pelvis	2	4.65	-
Volume (mL)	-	-	564
Intrathoracic	1	2.32	-
Volume (mL)	-	-	518
Neck	1	2.32	-
Volume (mL)	-	-	9
Intraperitoneal	0	-	-
**CT with IV contrast media**	30	97.78	-
**Arterial blush**	14	46.66	-
Unifocal	8	57.14	-
Multifocal	6	42.86	-
**PTE**	7	22.58	-
**Other thromboembolic ischaemic complications**	4	12.90	-

PTE, pulmonary thrombo-embolism; ISTH, International Society on Thrombosis and Haemostasis; IV, intravenous.

In 46.66% CT exams (14/30 performed with contrast medium), the authors found active arterial contrast extravasation (Table 1) A correlation was found between active haematomas and a unifocal blush (*p* = 0.000; *r* = 0.65) and with multifocal contrast extravasations (*p* = 0.002; *r* = 0.54). Significant correlations were observed between the presence of active arterial blush and male sex (*p* = 0.031; *r* = 0.39) and between the same event and a history of antiplatelet therapy for previous comorbidities (*p* = 0.022; *r* = 0.41). Finally, a significant inverse correlation was observed between bleeding with active contrast extravasation and the time elapsed from the start of anticoagulant treatment (*p* = 0.03; *r* = −0.43); haematomas with active bleeding occurred earlier.

### Laboratory

Result averages at admission and at CT diagnosis are listed in Table 2. There was a significant inverse correlation between major bleeding and haemoglobinaemia at the time of diagnosis (*p* = 0.004; *r* = −0.51), while a significant inverse correlation was found between platelet blood count at the time of diagnosis and the development of multiple haematomas (*p* = 0.027; *r* = −0.39). A significant inverse correlation was observed between the development of active bleeding and the activated partial thromboplastin time (aPTT) ratio on the day of hospitalisation (*p* = 0.05; *r* = −0.36).

**TABLE 2:** Patient characteristics, clinical features, treatment, outcome and laboratory tests.

Variable	Total (*n*)	%	Average (*n*)
**Patients**	31	-	-
Males	23	74.20	-
Females	8	25.80	-
**Age (years)**	-	-	71.10
**Length of hospitalisation (days)**	-	-	37.50
**ICU**	19	61.30	-
**Mechanical ventilation, prono-supination**	17	54.83	-
**CPAP**	14	41.17	-
**Anticoagulation**	31	100.00	
**Days since hospitalisation to the beginning of anticoagulation**	-	-	1.92
**Endovascular embolisation**	6	19.35	-
**No comorbidities**	1	3.22	-
**Single comorbidity**	2	6.45	-
**Two or more comorbidities**	28	90.32	-
**Deceased**	4	12.90	-
**Discharged (survived)**	27	87.10	-
**At hospital admission**	-	-	-
aPTT (nv 0.8–1.20 ratio)	-	-	1.04
Hb (nv M > 135 g/L, F > 120 g/L)	-	-	130.19
Platelets (nv 150–150–450 × 10^3^/μL)	-	-	225.93
PT/INR (nv 0.85–1.15)	-	-	1.43
D-dimer (nv 0–0.23)	-	-	0.78
**At first CT diagnosis**	-	-	-
aPTT (nv 0.8–1.20 ratio)	-	-	1.02
Hb (nv M > 135 g/L, F > 120 g/L)	-	-	95.54
Platelets (nv 150–150–450 × 10^3^/μL)	-	-	212.09
PT/INR (nv 0.85–1.15)	-	-	1.12
D-dimer (nv 0–0.23)	-	-	0.62

ICU, intensive care unit; CPAP, continuous positive airway pressure; aPTT, activated partial thromboplastin time; PT/INR, Prothrombin Time and International Normalised Ratio; nv, normal values; Hb, haemoglobin.

### Comorbidities

The presence and/or absence of comorbidities is listed in Table 2.

The most frequent comorbidities were hypertension in 27/31 cases (87.09%), body mass index (BMI) > 30 in 15/31 cases (48.38%) and heart disease in 15/31 cases (48.38%). Less frequent were dyslipidaemia in 12/31 cases (38.70%), type II diabetes in 8/31 cases (25.80%), non-COVID-19 respiratory disease in 5/31 cases (16.13%), neurological pathology in 4/31 cases (12.9%), liver disease in 3/31 cases (9.67%), chronic kidney disease in 2/31 cases (6.45%) and known chronic infectious diseases, rheumatological, haematological, oncological conditions and endocrinopathies in one case each.

The authors found a significant direct correlation between the presence of multiple comorbidities and arterial hypertension (*p* = 0.002; *r* = 0.52), while obesity was more significant in males (*p* = 0.018; *r* = 0.42). The inverse correlations between age and obesity (*p* = 0.000; *r* = −0.69) and age and type II diabetes (*p* = 0.01; *r* = −0.45) were very significant. A significant inverse correlation between the presence of multiple comorbidities and the time elapsed from hospitalisation to diagnosis (*p* = 0.046; *r* = −0.36) was also observed; in these patients, the haematomas developed earlier.

### Other complications

Of the 31 patients, 10 (32.25%) developed thromboembolic ischaemic complications during hospitalisation despite all being anticoagulated patients; 7/31 patients (22.58%) developed pubmonary thromboembolism (PTE) (Figures 3 and 6), in one case with deep vein thrombosis (DVT) (Figure 6), one subtotal splenic infarction (Figure 1), one subtotal renal ischaemia and one DVT.

### Treatment

Of the 31 patients, 17 (54.83%) underwent invasive ventilation manoeuvres with pronation and supination cycles, while 14/31 (47.17%) underwent C-PAP ventilation.

All patients received anticoagulant therapy during hospitalisation. Therapy started on average 1.92 days after admission from −2 to 17 days; one patient had already started treatment at home two days before admission. Of the 31 patients, 5 (16.12%) were already on anticoagulant therapy for previous pathologies, while 12/31 (38.7%) received the treatment upon hospital admission. Of the 31 patients, 13 (41.9%) were on antiplatelet therapy prior to admission.

In 25/31 cases (80.65%) the treatment of haematomas was conservative, while 6/31 patients (19.35%) underwent endovascular embolisation at the hub hospital; 2/6 (33.3%) later demised. The haematomas were not surgically evacuated in any of the cases. A significant direct correlation was found between embolisation and active contrast extravasation (*p* = 0.002; *r* = 0.54) and with the evidence of multifocal contrast blushes on CT (*p* = 0.001; *r* = 0.59).

### Outcome

During the review period, 1396 patients affected by COVID-19 were admitted to the authors’ centre (241/1396 [17.26%] in the ICU) of whom 25.14% (351/1396) died, (77/241 [31.95%] in the ICU). Among patients with spontaneous bleeding, there were 4/31 deaths (12.90%) from any cause. This equated to 1.13% (4/351) of all deaths of patients affected by COVID-19 and 5.2% (4/77) of ICU deaths. All four deceased patients had multiple comorbidities, were hospitalised in the ICU and had developed major bleeding.

These patients, two males and two females, with a mean age of 75.75 years (75 years, males; 76.5 years, females), who died at an average of 19.75 days after admission (range: 18–24), had received the CT diagnosis of haematoma on an average of 14.5 days after admission. Pulmonary embolism also occurred in one case and septic shock in another.

The mean volume of haematomas in deceased patients was 953.2 mL; in 3/4 cases (75%), the bleeding involved only one anatomical site; and in 3/4 cases (75%), the bleeding was active (in two cases with multifocal contrast blush). A significant inverse correlation was found between death and haemoglobin values on the day of admission (*p* = 0.024; *r* = −0.40): haemoglobin at admission was significantly lower in patients who later died.

The 27/31 surviving patients (87.1%, mean age 70.4 years) were discharged after an average hospital stay of 40.22 days (30.5 if hospitalised in the ward, 47.93 if in the ICU); 21/27 (77.8%) suffered major bleeding (4/21 treated with embolisation) and developed haematomas with a mean volume of 634.26 mL, diagnosed on average 17 days after admission.

The main imaging and population characteristics are summarised in Table 1 and Table 2.

## Discussion

### Role of anticoagulation in COVID-19 therapy and risk factors for haematomas

Anticoagulant treatment is recognised as a possible cause of muscle haematomas in adult patients unaffected by COVID-19.^5,6^ During the COVID-19 pandemic, in addition to the pulmonary and neurological manifestations, the alteration of the coagulation pathway soon appeared significant, which represented the rationale for the use of heparin.^5,7,8,9,10,11,12,13,14,15,16^ This alteration resulted both in the form of hypercoagulability^11,14,17^ with expressions of a thromboembolic nature (e.g. PTE, DVT), whose mechanism is still necessary to investigate,^13^ and in the form of haemorrhagic diathesis with depletion of coagulation factors, thrombocytopenia and hyperfibrinolysis.^14,18^

Among the risk factors for the development of bleeding in COVID-19 patients, contributing causes have been proposed, such as pronation manoeuvres, anticoagulant treatment, obesity, increased vascular fragility determined by the pro-inflammatory state, barotrauma from ventilation by C-PAP and cough with consequent increase in intra-abdominal pressure and arterial rupture.^17,19^ Many of these risk factors coincide with those that have been documented in this article.

Godier and colleagues observed that bleeding occurred in the hyperinflammation reduction phase typical of these patients, as evidenced by the reduction of fibrinogen and D-dimer.^20^ The biphasic coagulative alterations proposed by them, firstly pro-thrombotic and subsequently haemorrhagic in the phase of resolution of the inflammation, could be useful in explaining the average 16.7 days which elapsed from hospitalisation to diagnosis in our population. Other authors have also highlighted the development of the complication at about two weeks.^21,22^ As proposed by Kessler et al., it could therefore be reasonable to reduce anticoagulant treatment after 10–14 days in patients with favourable clinical progress.^23^

### Epidemiology

Qiu et al. reported that COVID-19 itself represents a risk factor for bleeding by detecting bleeding in 35% of a cohort of these patients versus 10% in a cohort of patients with community-acquired pneumonia.^24^

A frequency of bleeding complications ranging from 4.8% to 8% with 3.5% major bleeding has been reported in patients with COVID-19.^9,25,26^ However, these frequencies are widely variable. Fraissé et al. reported a bleeding rate of 21% in their critically ill patients, 84% after anticoagulation at therapeutic doses.^27^ In two different papers, Al-Samkari et al. reported an overall bleeding rate of 4.8% with 2.3% major bleeding^9^ while in critically ill patients they reported a 2.8% rate of major bleeding approximately two weeks after admission to the hospital ICU.^28^ Demelo-Rodriguez et al. found less frequent bleeding in patients admitted to the non-intensive ward compared with those in intensive care.^29^ In our case study 61.29% of bleeding (19/31) occurred in ICU patients compared with 38.71% (12/31) in patients in other wards, although this difference did not show significance.

Lucatelli et al.^1^ reported a predominance of this complication in males (68.00% vs 74.20% in this study) and multifocal bleeding in 68.40% of cases, which is higher than the 29.03% in this study. In the authors’ ICU, the incidence of complication was 7.88% (19/241) with 7.46% (18/241) major bleeding, compared with an overall incidence of 2.22% (31/1396) with 2.07% (29/1396) major bleeding; the data are substantially comparable to that reported by other authors.^30,31^

### Symptoms and imaging

Muscular haematomas are blood spills in a muscle group that can remain contained by the fascial plane or spread to adjacent spaces (e.g. peritoneum) and are spontaneous if not associated with trauma.^32^ Symptoms of muscle haematomas are of variable magnitude: local (e.g. skin hypersensitivity, peritoneal signs in the abdominal wall or retroperitoneal haematomas) and general (e.g. tachycardia, hypotension, pallor), while complications include compartment syndrome, superinfections, compressive neuropathies and diffusion in adjacent spaces (e.g. haemoperitoneum).^32,33^

CT imaging with contrast medium is the gold standard for diagnosis and allows for defining the topography and size of the bleeding, highlighting compression complications, showing active contrast extravasation, providing useful indications to the interventionist on the bleeding vessel, as well as evaluating the possible coexistence of thromboembolic or ischaemic manifestations.^8,13,26,32,34^

Dohan and colleagues argued that the presence of active bleeding is related to a greater severity of the clinical picture.^32^ In 75% (3/4) of our deceased patients, CT imaging detected active bleeding versus 40.7% (11/27) in the surviving patients, although this difference did not have statistical significance.

The most commonly represented bleeding site in our case study was the retroperitoneum. In their case report, Nakamura et al. recalled the Lenk triad (haemodynamic shock, palpable mass, severe pain) as a possible manifestation of these haematomas^26^ while some authors have shown that retroperitoneal haematomas are associated with high mortality and can manifest themselves with haemorrhagic shock and are often underdiagnosed, especially in sedated and intubated patients.^11,34,35^ Vergori et al. highlighted a higher incidence of retroperitoneal haemorrhages in COVID-19 patients with 7.6 cases/1000 hospitalisations (12.18 cases/1000 hospitalisations in this series) versus 3.8 cases/1000 reported in critically ill non-COVID-19 patients.^11,36^

As in some cases in our study, Benazzi et al. reported pulmonary embolism in one of their two cases,^17^ Dennison et al. reported the case of a patient with splenic ischaemia, abdominal wall haemorrhage and mesenteric vessel infarction,^13^ while concomitance of DVT and haemorrhage has been reported by other authors.^19,37^

### Laboratory

Shah et al. observed that prothrombin time is a poor predictor of bleeding,^25^ Dohan et al. found International Normalized Ratio (INR) values in the therapeutic range in many cases^32^ while Sottilotta et al. reported that there are no specific laboratory predictors for the onset of haematomas.^38^ Lucatelli and colleagues highlighted the role of COVID-19-induced thrombocytopenia as a cause for an increased risk of major bleeding during treatment with therapeutic dose heparin.^1^ In the work of Al-Samkari et al., the D-dimer value at hospitalisation was considered predictive of the risk of bleeding, thrombosis, clinical severity and death while the prolongation of the prothrombin time was associated with a reduction in survival and a worse clinical picture.^9^

In this case study, although the mean INR, aPTT and blood platelet count values were normal at the time of haematoma diagnosis, more active bleeding was observed in patients with lower aPTT values at admission and multiple bleeding in patients with lower blood platelet count values at diagnosis. The mean D-dimer value was high both at admission and at diagnosis, although this was not statistically significant. Patients with a poor outcome had lower haemoglobin on admission.

### Treatment

As there are no guidelines for the treatment of the complication, treatment must be multidisciplinary and ‘tailored’ to the individual patient.^11,13,39^ However, Lucatelli et al. supported the endovascular approach compared to conservative management.^18^ Riu and colleagues highlighted some severity criteria such as active blood extravasation, retroperitoneal site, conspicuous size, haematoma wall rupture, reduction in haemoglobin greater than 2 g/dL or 3 g/dL, need for repeated transfusions and presence of thromboembolic complications with difficulty in interrupting anticoagulation.^39^

Dohan and colleagues proposed the surgical option in cases of nerve compression or cutaneous ischaemia, however, highlighting frequent relapse with this approach. They underlined that small intrafascial haematomas can self-resolve, suggesting an endovascular approach for voluminous active haematomas.^32^ Interestingly, while highlighting how heparin-induced bleeding tends to self-resolve upon discontinuation of anticoagulant treatment, Lucatelli et al. decided not to discontinue this therapy, considering it necessary in patients with COVID-19, given its association with an increase in survival in critically ill patients.^1,40,41,42^

In this case study, 80.65% of patients (25/31) were managed conservatively. Among the six embolised patients, two died (33.33%) versus two (8.00%) patients who died among the 25 patients managed conservatively; however, this difference was not statistically significant.

### Outcome

Mattioli et al. believed that the risk of haemorrhagic events could be underestimated as the focus appears more on the pro-thrombotic side of the alterations in the coagulative cascade, possibly also because of the lower lethality of the haemorrhagic complications.^14,35^

Al-Samkari et al. observed a 28-day mortality rate of 62.2% among critically ill patients with bleeding complications compared with 38.2% among patients with venous thromboembolic complications; however, noting a frequency of bleeding complications of approximately half than that of venous thromboembolic complications.^28^

The overall mortality among patients admitted for COVID-19 at the authors’ centre was 25.14% (351/1396) and 31.95% (77/241) among those admitted into the ICU, substantially in line with the overall mortality in ICU patients of 31.6% reported by Shah et al.,^25^ In our cohort of bleeding patients, mortality was 12.90% (4/31) representing only 1.13% of all deaths among patients with COVID-19 (4/351).

Furthermore, unlike Godier et al. who reported a mortality of 50.00% among critically ill patients with bleeding complications (vs 37.00% in patients without bleeding),^20^ this case study found lower mortality among patients bleeding in the ICU (21.05%, 4/19) versus 32.88% (73/222) critically ill non-bleeding patients; this difference was not statistically significant.

## Conclusion

Although some authors have expressed doubts about anticoagulant treatment in patients with COVID-19,^43,44,45^ the bleeding complication in this study did not significantly affect the outcome. Bleeding, major in most cases, occurred in anticoagulated patients, especially males with multiple comorbidities (e.g. obesity and heart disease), aged between 60 and 79 years and mainly appeared in a single anatomical region (especially retroperitoneal), with the most voluminous in the chest wall. The complication was diagnosed on average 16.7 days after admission and occurred predominantly in critically ill patients undergoing invasive ventilation and pronation-supination cycles. In just under half of the cases, the haematomas were active and mainly with single contrast extravasation. In addition, taking into account the start of anticoagulation, haematomas with active bleeding had an earlier onset than those without contrast blush. Major bleeding was also experienced earlier in the presence of multiple morbidity and the vast majority of patients were treated conservatively and survived.

At COVID-19 hospital centres, it is advisable that there is knowledge of such a complication for which CT imaging is essential for proper management.

### Limitations and bias

The study is retrospective in nature and in the absence of a control group it is difficult to state the real impact of the complication on the outcome. Anticoagulant treatment is another bias; moreover the authors did not stratify the population based on the dose of anticoagulant administered. In patients who developed multiple haematomas, we only reported the date of the first diagnostic CT scan and only bleeding diagnosed by CT was recorded. The history of non-iatrogenic trauma found in two patients could be a confounding factor.

## References

[1] Lucatelli P, De Rubeis G, Citone M, et al. Heparin-related major bleeding in Covid-19-positive patient: Perspective from the outbreak. Cardiovasc Intervent Radiol. 2020;43:1216–1217. 10.1007/s00270-020-02532-3 32468143 PMC7255445

[2] Brandi N, Ciccarese F, Rimondi MR, et al. An imaging overview of COVID-19 ARDS in ICU patients and its complications: A pictorial review. Diagnostics. 2022;12(4):846. 10.3390/diagnostics12040846 35453894 PMC9032937

[3] Gabelloni M, Faggioni L, Cioni D, et al. Extracorporeal membrane oxygenation (ECMO) in COVID-19 patients: A pocket guide for radiologists. La Radiol Med. 2022;127:369–382. 10.1007/s11547-022-01473-w PMC8918086 35279765

[4] Schulman S, Kearon C. Definition of major bleeding in clinical investigations of antihemostatic medicinal products in non-surgical patients. J Thromb Haemost. 2005;3(4):692–694. 10.1111/j.1538-7836.2005.01204.x 15842354

[5] Rogani S, Calsolaro V, Franchi R, Calabrese AM, Okoye C, Monzani F. Spontaneous muscle hematoma in older patients with COVID-19: Two case reports and literature review. BMC Geriatr. 2020;20(1):539. 10.1186/s12877-020-01963-4 33353545 PMC7755066

[6] Nourbakhsh E, Anvari R, Nugent K. Abdominal wall hematomas associated with low-molecular-weight heparins: An important complication in older adults. J Am Geriatr Soc. 2011;59(8):1543–1545. 10.1111/j.1532-5415.2011.03529.x 21848819

[7] Thachil J, Tang N, Gando S, et al. ISTH interim guidance on recognition and management of coagulopathy in COVID-19. J Thromb Haemost. 2020;18(5):1023–1026. 10.1111/jth.14810 32338827 PMC9906133

[8] Patel I, Akoluk A, Douedi S, et al. Life-threatening psoas hematoma due to retroperitoneal hemorrhage in a COVID-19 patient on enoxaparin treated with arterial embolization: A case report. J Clin Med Res. 2020;12(7):458–461. 10.14740/jocmr4256 32655742 PMC7331864

[9] Al-Samkari H, Karp Leaf RS, Dzik WH, et al. COVID-19 and coagulation: Bleeding and thrombotic manifestations of SARS-CoV-2 infection. Blood. 2020;136(4):489–500. 10.1182/blood.2020006520 32492712 PMC7378457

[10] Wool GD, Miller JL. The impact of COVID-19 disease on platelets and coagulation. Pathobiology. 2021;88(1):15–27. 10.1159/000512007 33049751 PMC7649697

[11] Vergori A, Pianura E, Lorenzini P, et al. Spontaneous ilio-psoas haematomas (IPHs): A warning for COVID-19 inpatients. Ann Med. 2021;53(1):295–301. 10.1080/07853890.2021.1875498 33491498 PMC7877978

[12] Bargellini I, Cervelli R, Lunardi A, et al. Spontaneous bleedings in COVID-19 patients: An emerging complication. Cardiovasc Intervent Radiol. 2020;43:1095–1096. 10.1007/s00270-020-02507-4 32419077 PMC7231527

[13] Dennison JJ, Carlson S, Faehling S, Phelan H, Tariq M, Mubarik A. Splenic infarction and spontaneous rectus sheath hematomas in COVID-19 patient. Radiol Case Rep. 2021;16(5):999–1004. 10.1016/j.radcr.2021.02.016 33619439 PMC7881734

[14] Dorgalaleh A. Bleeding and bleeding risk in COVID-19. Semin Thromb Hemost. 2020;46(7):815–818. 10.1055/s-0040-1713434 32512587 PMC7645827

[15] Tang N, Bai H, Chen X, Gong J, Li D, Sun Z. Anticoagulant treatment is associated with decreased mortality in severe coronavirus disease 2019 patients with coagulopathy. J Thromb Haemost. 2020;18(5):1094–1099. 10.1111/jth.14817 32220112 PMC9906401

[16] Marietta M, Ageno W, Artoni A, et al. COVID-19 and haemostasis: A position paper from Italian Society on Thrombosis and Haemostasis (SISET). Blood Transfus [serial online]. 2020 [cited 2022 Jan 19];18(3):167–169. Available from: https://pubmed.ncbi.nlm.nih.gov/32281926/10.2450/2020.0083-20 PMC7250682 32281926

[17] Benazzi D, Antonicelli V, Presciuttini B, et al. COVID-19 and hemorrhagic complications: Pectoral hematoma. Ital J Emerg Med. 2021;10(1):6–10. 10.23736/S2532-1285.21.00044-6

[18] Lucatelli P, Rocco B, Nardis PG, et al. Bleeding in COVID patients: What we have understood so far. Cardiovasc Intervent Radiol. 2021;44:666–668. 10.1007/s00270-021-02775-8 33511426 PMC7843001

[19] Conti CB, Henchi S, Coppeta GP, Testa S, Grassia R. Bleeding in COVID-19 severe pneumonia: The other side of abnormal coagulation pattern? Eur J Intern Med. 2020;77:147–149. 10.1016/j.ejim.2020.05.002 32414639 PMC7203058

[20] Godier A, Clausse D, Meslin S, et al. Major bleeding complications in critically ill patients with COVID-19 pneumonia. J Thromb Thrombolysis. 2021;52(1):18–21. 10.1007/s11239-021-02403-9 33646501 PMC7919235

[21] Boira I, Esteban V, Vañes S, Castelló C, Celis C, Chiner E. Major bleeding complications in COVID-19 patients. Cureus. 2021;13(8):e16816. 10.7759/cureus.16816 34522476 PMC8425135

[22] Conci S, Ruzzenente A, Donadello K, et al. Haemodynamic instability in a critically ill patient with covid-19 pneumonia: Searching over the chest – Report of a clinical case and mini-review of the literature. Case Rep Imag Surg. 2020;3(2-3). 10.15761/CRIS.1000141

[23] Kessler C, Stricker H, Demundo D, et al. Bleeding prevalence in COVID-19 patients receiving intensive antithrombotic prophylaxis. J Thromb Thrombolysis. 2020;50:833–836. 10.1007/s11239-020-02244-y 32803737 PMC7427750

[24] Qiu C, Li T, Wei G, et al. Hemorrhage and venous thromboembolism in critically ill patients with COVID-19. SAGE Open Med. 2021;9:205031212110201. 10.1177/20503121211020167 PMC8170290 34104439

[25] Shah A, Donovan K, McHugh A, et al. Thrombotic and haemorrhagic complications in critically ill patients with COVID-19: A multicentre observational study. Crit Care. 2020;24(1):561. 10.1186/s13054-020-03260-3 32948243 PMC7499016

[26] Nakamura H, Ouchi G, Miyagi K, et al. Case report: Iliopsoas hematoma during the clinical course of severe COVID-19 in two male patients. Am J Trop Med Hyg. 2021;104(3):1018–1021. 10.4269/ajtmh.20-1507 33534775 PMC7941852

[27] Fraissé M, Logre E, Pajot O, Mentec H, Plantefève G, Contou D. Thrombotic and hemorrhagic events in critically ill COVID-19 patients: A French monocenter retrospective study. Crit Care. 2020;24:275. 10.1186/s13054-020-03025-y 32487122 PMC7265664

[28] Al-Samkari H, Gupta S, Leaf RK, et al. Thrombosis, bleeding, and the observational effect of early therapeutic anticoagulation on survival in critically ill patients with covid-19. Ann Intern Med. 2021;174(5):622–632. 10.7326/L21-0148 33493012 PMC7863679

[29] Demelo-Rodriguez P, Farfán-Sedano AI, Pedrajas JM, et al. Bleeding risk in hospitalized patients with COVID-19 receiving intermediate- or therapeutic doses of thromboprophylaxis. J Thromb Haemost. 2021;19(8):1981–1989. 10.1111/jth.15400 34018658 PMC8237051

[30] Dalager-Pedersen M, Christian Lund L, Mariager T, et al. Venous thromboembolism and major bleeding in patients with COVID-19: A nationwide population-based cohort study. Clin Infect Dis. 2021;73(12):2283–2293. 10.1093/cid/ciab003 33400771 PMC7929126

[31] Abate V, Casoria A, Rendina D, et al. Spontaneous muscle hematoma in patients with COVID-19: A systematic literature review with description of an additional case series. Semin Thromb Hemost. 2021;48(1):100–108. 10.1055/s-0041-1732370 34388842

[32] Dohan A, Darnige L, Sapoval M, Pellerin O. Spontaneous soft tissue hematomas. Diagn Intervent Imag. 2015;96(7–8):789–796. 10.1016/j.diii.2015.03.014 26066549

[33] Ramani SL, Samet J, Franz CK, et al. Musculoskeletal involvement of COVID-19: Review of imaging. Skelet Radiol. 2021;50:1763–1773. 10.1007/s00256-021-03734-7 PMC7889306 33598718

[34] Mondie C, Maguire NJ, Rentea RM. Retroperitoneal hematoma. Treasure Island, FL: StatPearls Publishing, 2021. 32644354

[35] Mattioli M, Benfaremo D, Fustini E, Gennarini S. Atypical spontaneous hematomas in a patient with severe coronavirus disease 2019 (COVID-19). Semin Thromb Hemost. 2020;46(7):856–858. 10.1055/s-0040-1715092 32877960 PMC7645830

[36] Artzner T, Clere-Jehl R, Schenck M, et al. Spontaneous ilio-psoas hematomas complicating intensive care unit hospitalizations. PLoS One. 2019;14(2):e0211680. 10.1371/journal.pone.0211680 30794573 PMC6386274

[37] Erdinc B, Raina JS. Spontaneous retroperitoneal bleed coincided with massive acute deep vein thrombosis as initial presentation of COVID-19. Cureus. 2020;12(8):e9772. 10.7759/cureus.9772 32953290 PMC7491698

[38] Sottilotta G, Mangano C, Basile R, et al. Iliopsoas hematoma in patients with COVID-19 on low-molecular-weight heparin treatment. SAGE Open Med Case Rep. 2021;9. 10.1177/2050313X211016991 PMC8161851 34104443

[39] Riu P, Albarello F, Di Stefano F, et al. Management of spontaneous bleeding in covid-19 inpatients: Is embolization always needed? J Clin Med. 2021;10(18):4119. 10.3390/jcm10184119 34575230 PMC8469448

[40] Paranjpe I, Fuster V, Lala A, et al. Association of treatment dose anticoagulation with in-hospital survival among hospitalized patients with COVID-19. J Am Coll Cardiol. 2020;76(1):122–124. 10.1016/j.jacc.2020.05.001 32387623 PMC7202841

[41] Musoke N, Lo KB, Albano J, et al. Anticoagulation and bleeding risk in patients with COVID-19. Thromb Res. 2020;196:227–230. 10.1016/j.thromres.2020.08.035 32916565 PMC7444469

[42] Nadkarni GN, Lala A, Bagiella E, et al. Anticoagulation, bleeding, mortality, and pathology in hospitalized patients with COVID-19. J Am Coll Cardiol. 2020;76(16):1815–1826. 10.1016/j.jacc.2020.08.041 32860872 PMC7449655

[43] Chan NC, Weitz JI. COVID-19 coagulopathy, thrombosis, and bleeding. Blood. 2020;136(4):381–383. 10.1182/blood.2020007335 32702124 PMC7378461

[44] Santoro F, Núñez-Gil IJ, Viana-Llamas MC, et al. Anticoagulation therapy in patients with coronavirus disease 2019: Results from a multicenter international prospective registry (health outcome predictive evaluation for corona virus disease 2019 [HOPE-COVID19]). Crit Care Med. 2021;49(6):e624–e633. 10.1097/CCM.0000000000005010 33861553

[45] Pesavento R, Ceccato D, Pasquetto G, et al. The hazard of (sub)therapeutic doses of anticoagulants in non-critically ill patients with Covid-19: The Padua province experience. J Thromb Haemost. 2020;18(10):2629–2635. 10.1111/jth.15022 32692874 PMC7404507

